# Long noncoding RNA HOTTIP mediates SRF expression through sponging miR‐150 in hepatic stellate cells

**DOI:** 10.1111/jcmm.14068

**Published:** 2018-12-08

**Authors:** Jianjian Zheng, Yuqing Mao, Peihong Dong, Zhiming Huang, Fujun Yu

**Affiliations:** ^1^ Key Laboratory of Diagnosis and Treatment of Severe Hepato‐Pancreatic Diseases of Zhejiang Province The First Affiliated Hospital of Wenzhou Medical University Wenzhou China; ^2^ Department of Gastroenterology Shanghai First People's Hospital School of Medicine Shanghai Jiao Tong University Shanghai China; ^3^ Department of Infectious Diseases The First Affiliated Hospital of Wenzhou Medical University Wenzhou China; ^4^ Departments of Gastroenterology and Hepatology The First Affiliated Hospital of Wenzhou Medical University Wenzhou China; ^5^ Department of Gastroenterology Songjiang Hospital Affiliated Shanghai First People's Hospital Shanghai Jiao Tong University Shanghai China

**Keywords:** competing endogenous RNA, hepatic stellate cell, HOTTIP, miR‐150

## Abstract

HOXA transcript at the distal tip (HOTTIP) has been shown to be up‐regulated in a variety of cancers and is identified as an oncogenic long noncoding RNA. However, the biological role of HOTTIP in liver fibrosis is unclear. Here, we reported that HOTTIP was specifically overexpressed in activated hepatic stellate cells (HSCs). HOTTIP knockdown suppressed the activation and proliferation of HSCs. Luciferase reporter assay showed that HOTTIP and serum response factor (SRF) were targets of miR‐150. RNA binding protein immunoprecipitation assay indicated the interaction between miR‐150 and HOTTIP. Further study revealed that HOTTIP increased SRF expression as a competing endogenous RNA for miR‐150, thus prompting HSC activation. Taken together, we provide a novel HOTTIP‐miR‐150‐SRF signalling cascade in liver fibrosis.

## 1 INTRODUCTION

Liver fibrosis is a wound‐healing response to chronic liver injury.^1^ During chronic injury, the liver parenchyma is gradually replaced with excessive extracellular matrix, eventually causing disruption of liver architecture and the loss of liver function. Hepatic stellate cell (HSC) activation is regarded as the important event in the progression of liver fibrosis.^2^, ^3^ Recently, it has been reported that many noncoding RNAs have been linked to HSC activation.^4^, ^5^ However, the function and underlying mechanism of noncoding RNAs in liver fibrosis is far from being fully elucidated.

MicroRNAs (miRNAs) have been reported to be implicated in many cell functions, such as proliferation, differentiation, development, and apoptosis.^6^, ^7^ miRNAs are differentially expressed during the course of liver fibrosis. For instance, the increased expression of miR‐125a and miR‐34a as well as the decreased expression of miR‐181, miR‐200a, and miR‐29b are observed in fibrotic livers.^8^, ^9^, ^10^, ^11^, ^12^ miR‐150 is reduced in activated HSCs and its mimics decreases HSC activation through inhibiting c‐myb expression.^13^ Our previous data also showed that miR‐150 can hinder the activation of HSCs via inhibition of Sp1 and Col4A4.^14^ Whether there are other targets of miR‐150 remains largely unknown.

Increasing evidence indicates that HOTTIP is overexpressed in prostate cancer, lung cancer, and pancreatic cancer.^15^, ^16^, ^17^ HOTTIP knockdown impedes cell viability, proliferation, invasion, and angiogenesis in human cancer cells,^18^, ^19^ therefore it is been identified as an oncogenic long noncoding RNA (lncRNA). In addition, HOTTIP overexpression is in association with poor prognosis in cancers.^20^, ^21^ HOTTIP expression is regulated by miR‐125, miR‐192, and miR‐204.^22^, ^23^ However, whether HOTTIP can modulate miRNAs has not been investigated. Many studies have showed that lncRNAs can act as natural sponge to modulate target expression by sequestering miRNAs.^24^, ^25^ However, it is unclear whether HOTTIP may also have such a role to link miRNA and its target in liver fibrosis.

In the present study, our results showed that HOTTIP is specifically in activated HSCs. HOTTIP silencing can hinder activation and proliferation of HSCs. Further study revealed that HOTTIP acts as a ceRNA to enhance serum response factor (SRF) expression by sequestering miR‐150, thus promoting the activation of HSCs.

## 2 MATERIALS AND METHODS

### 2.1 Animals

Male C57BL/6J mice (20 ± 2 g) were bred in the animal house without specific pathogen. All experiments in this study were approved by the Animal Ethics Committee of Wenzhou Medical University. Mouse liver fibrosis was set up via a biweekly intraperitoneal injection with a 10% solution of carbon tetrachloride (CCl_4_, Sigma‐Aldrich, St. Louis, MO, USA) diluted in olive oil for 8 weeks.

### 2.2 Cell culture

Primary mouse HSCs were obtained by pronase/collagenase perfusion solution plus density gradient centrifugation, as previously detailed.^26^ The purity of the isolated HSCs was evaluated based on immunocytochemical staining for a‐SMA and the purity was in excess of 95%. Mouse hepatocytes were obtained using collagenase perfusion technique as previously described.^27^ Cells were cultured in Dulbecco's modified Eagle's medium including 10% foetal bovine serum, streptomycin (100 g/mL), and penicillin (100 units/mL). The cells were incubated at 37°C in a humidified incubator of 5% CO_2_.

### 2.3 Adenovirus production

The adenoviral vector containing HOTTIP DNA sequence (Ad‐HOTTIP‐wt), the adenoviral vector containing mutated (predicted miR‐150 binding sites) HOTTIP DNA sequence (Ad‐HOTTIP‐mut), the adenoviral vector containing HOTTIP shRNA (Ad‐shHOTTIP), the adenoviral vector containing SRF shRNA (Ad‐shSRF), and the negative control were purchased from Shanghai Genechem (Shanghai, China).

### 2.4 Plasmid construction

To generate pmirGLO‐SRF‐wt, pmirGLO‐HOTTIP‐wt, and pcDNA‐HOTTIP‐wt, mouse SRF 3′UTR and HOTTIP cDNA were amplified by PCR and then subcloned into pmirGLO plasmid or pcDNA3.1 (Life Technologies, Carlsbad, CA, USA). pmirGLO‐HOTTIP‐mut and pmirGLO‐SRF‐mut were generated by the site‐directed mutagenesis kit (Stratagene, La Jolla, CA, USA). The mutated sequences were located in the predicted miR‐150 binding sites (Figures 3B and 4A). The constructs were validated by DNA sequencing.

### 2.5 Immunofluorescence and Immunohistochemistry

Cells were fixed with an acetic acid:ethanol (1:3) solution, permeabilized in 0.1% PBS‐Tween, and then blocked with 5% goat and horse serum/PBS. Then cells were treated with mouse primary antibodies against α‐SMA and type I collagen (Abcam, Cambridge, UK) overnight at 4°C, followed by Alexa Fluor 568‐labeled rabbit anti‐mouse IgG (Life Technologies). Images were examined with a Carl Zeiss LSM710 confocal microscope (Carl Zeiss AG, Jena, Germany). After liver sections were dewaxed, dehydrated, and subjected to antigen retrieval, the samples were treated overnight at 4°C with mouse a‐SMA antibody, followed by a biotinylated secondary antibody. The α‐SMA expression was visualized by DAB staining.

### 2.6 Quantitative real‐time PCR (qRT‐PCR)

The total RNA was isolated from HSCs and liver tissues using TRIzol reagent (Life Technologies) in accordance with the manufacturer's instructions. qRT‐PCR was performed on a 7900HT Fast Real‐Time PCR system (Applied Biosystems, Foster City, CA, USA) with KAPA SYBR FAST qPCR kit master mix (2×) universal (KapaBiosystems, Boston, MA, USA). The relative expression of mRNAs, miRNAs, and lncRNAs were calculated through the 2^−ΔΔCt^ method. All primers are shown in Table 1.

**Table 1:** List of primer sequences

Mouse	Forward	TGCACCACCAACTGCTTAG
GAPDH	Reverse	GGATGCAGGGATGATGTTC
Mouse	Forward	CCTGGCAAAGACGGACTCAAC
Col1A1	Reverse	GCTGAAGTCATAACCGCCACTG
Mouse	Forward	TCCCTGGAGAAGAGCTACGAACT
α‐SMA	Reverse	AAGCGTTCGTTTCCAATGGT
Mouse	Forward	GAACTCCAACTGCATCAC
HOTTIP	Reverse	CTCGGCACCATCTAAATCACTC
Mouse	Forward	TTTGCCACCCGCAAACTG
SRF	Reverse	GACACCTGGTAGGTGAGATCTG
Mouse	Forward	CTCGCTTCGGCAGCACA
U6	Reverse	AACGCTTCACGAATTTGCGT
Mouse	Forward	ACACTCCAGCTGGGTCTCCCAACCCTTGTA
miR‐150	Reverse	TGGTGTCGTGGAGTCG
	RT	CTCAACTGGTGTCGTGGAGTCGGCAATT
		CAGTTGAGCACTGGTA

### 2.7 Luciferase reporter gene assay

HEK293T cells were incubated in a 24‐well plate for 24 hours. Then, cells in each well were cotransfected with pmirGLO plasmid, miR‐150 mimics, together with or without pcDNA‐HOTTIP (wt) or pcDNA‐HOTTIP (mut) using Lipofectamine 2000 (Life Technologies). Cells were collected 48 hours after transfection and the value of relative luciferase activity was measured by the Dual‐Luciferase Reporter Assay System (Promega, Madison, WI, USA).

### 2.8 RNA binding protein immunoprecipitation (RIP) assay

RIP experiment was performed using the EZ‐Magna RIP kit (Millipore, Billerica, MA, USA). After HSCs were lysed with RIP lysis buffer, the cells were incubated with anti‐Argonaute‐2 (Ago2) antibody or Isotype‐matched IgG. The samples were immunoprecipitated with proteinase k and RNA was isolated by TRIzol reagent. The quantification of HOTTIP and miR‐150 was analysed by qRT‐PCR.

### 2.9 Cell proliferation assay

HSCs were transduced with Ad‐shHOTTIP for 48 hours. Then the cells were incubated with 5‐Ethyny‐2′‐deoxyuridine (EdU) for 12 hours. Cell proliferation was examined using an EdU detection kit (Beyotime Biotechnology, Jiangsu, China) in keeping with the manufacturer's instructions.

### 2.10 Western blot

Proteins were isolated using a RIPA lysis buffer (Beyotime Biotechnology) and subjected to SDS‐PAGE. After blocking, the membrane was incubated with the primary antibodies (β‐actin, type I collagen, and α‐SMA, Abcam) and the secondary antibodies (Li‐Cor Biosciences Inc., Lincoln, NE, USA). Signals were examined using an Odyssey infrared scanner (Li‐Cor Biosciences Inc.).

### 2.11 Statistical analysis

The results were expressed as the mean ± SD. Differences between groups were compared using a Student's *t* test or one‐way analysis of variance. *P* < 0.05 was considered statistically significant. All statistical analyses were performed using SPSS 13.0 (IBM, Armonk, NY, USA).

## 3 RESULTS

### 3.1 HOTTIP is up‐regulated in activated HSCs

**Figure 1:**
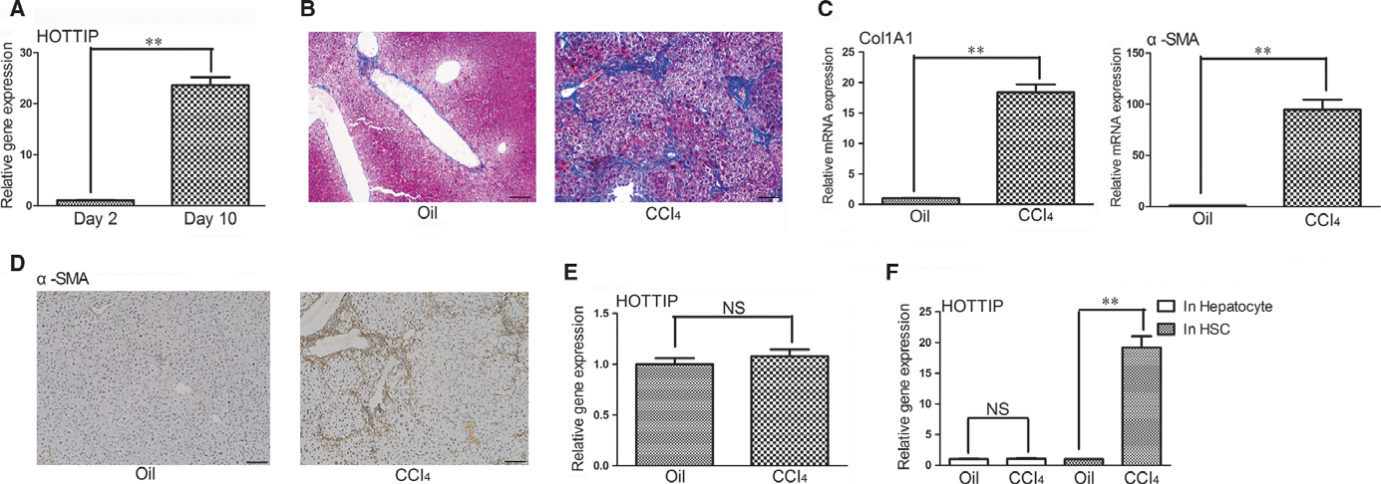
HOTTIP is specifically up‐regulated in activated HSCs. (A) Expression of HOTTIP was detected in primary HSCs at day 2 and day 10. (B) CCl_4_‐induced liver fibrosis model was analysed by Masson staining. Scale bars, 100 μm. (C) Expression of Col1A1 and α‐SMA was detected in livers of mice treated with CCl_4_ or oil. (D) The protein expression of α‐SMA was detected by an immunohistochemical method. Scale bars, 100 μm. (E) Expression of HOTTIP was detected in livers of mice treated with CCl_4_ or oil. (F) Expression of HOTTIP was detected in hepatocytes and HSCs isolated from oil or CCl_4_‐treated mice. Results are expressed as means ± SD, **P < 0.01

To explore the role of HOTTIP in liver fibrogenesis, we first analysed the expression of HOTTIP in HSCs. Primary quiescent HSCs were isolated from normal male C57BL/6J mice and cultured, which mimicks the in vivo activation process. qRT‐PCR results showed that HOTTIP expression at day 10 was increased by 22.6‐fold compared with that at day 2 (Figure 1A). Then we further analysed whether the alteration of HOTTIP exists in a mouse liver fibrosis model. Masson staining was performed to evaluate the degree of liver fibrosis (Figure 1B). The results of qRT‐PCR indicated that the mRNA expression of Col1A1 and α‐SMA were dramatically up‐regulated in CCl_4_‐treated mice compared with oil‐treated mice (Figure 1C). Immunohistochemistry results showed that the protein expression of α‐SMA in CCl_4_‐treated mice was increased compared with oil‐treated mice. However, the expression of HOTTIP was unaltered upon CCl_4_ treatment (Figure 1E).

Based on the above findings, we deduced that HOTTIP may be specifically increased in HSCs during liver fibrosis. Therefore, we analysed the expression of HOTTIP in primary HSCs and hepatocytes isolated from CCl_4_‐treated mice. Expectedly, HOTTIP expression was markedly up‐regulated in HSCs isolated from CCl_4_‐treated mice compared with those from oil‐treated mice, whereas we failed to find a remarkable up‐regulation of HOTTIP in hepatocytes (Figure 1F). In conclusion, these results showed that HOTTIP is specifically dysregulated in HSCs during experimental fibrogenesis.

### 3.2 Activation and proliferation of HSCs is negatively regulated by HOTTIP knockdown

**Figure 2:**
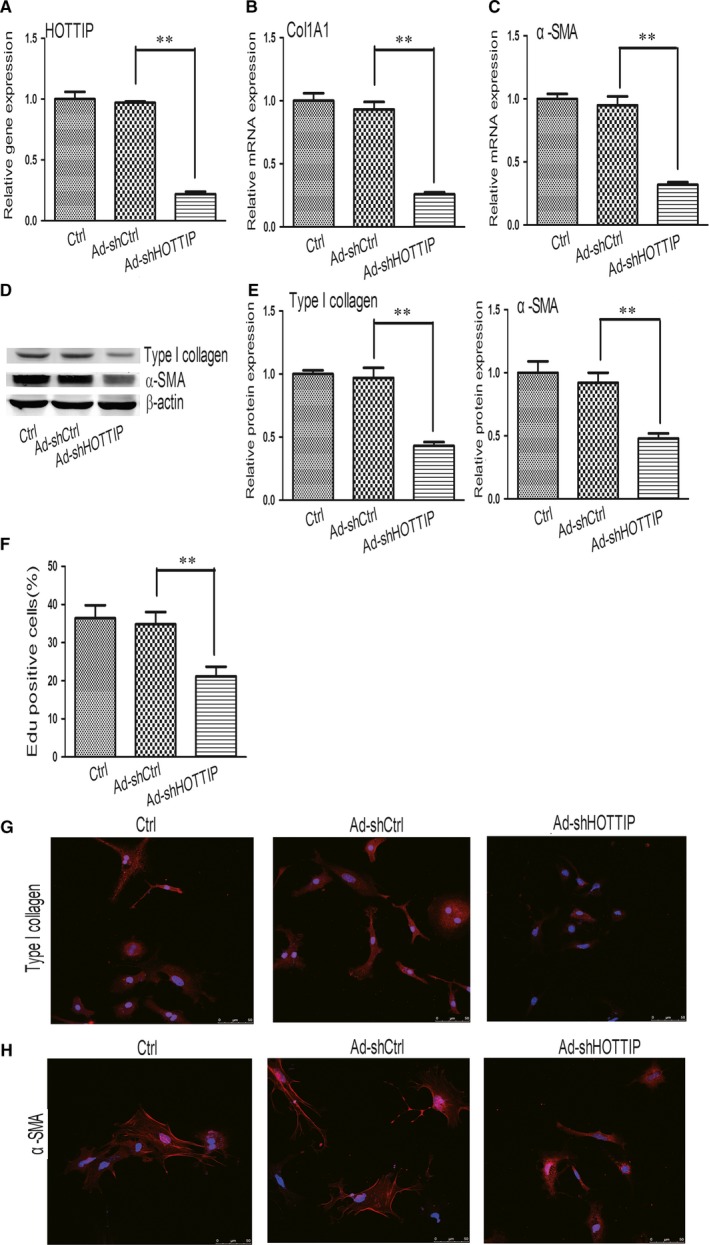
Knockdown of HOTTIP suppresses activation and proliferation of HSCs. (A) HOTTIP gene expression was detected in primary HSCs after Ad‐shHOTTIP treatment. (B) The mRNA expression of Col1A1 was detected in primary HSCs after Ad‐shHOTTIP treatment. (C) The mRNA expression of α‐SMA was detected in primary HSCs after Ad‐shHOTTIP treatment. (D, E) The protein expression of type I collagen and α‐SMA was detected in primary HSCs after Ad‐shHOTTIP treatment. (F) HSC proliferation was detected using an Edu incorporation assay in primary HSCs after Ad‐shHOTTIP treatment. (G) The protein expression of type I collagen in primary HSCs after Ad‐shHOTTIP treatment was analysed using an immunocytochemical method. (H) The protein expression of α‐SMA in primary HSCs after Ad‐shHOTTIP treatment was analysed using an immunocytochemical method. Results are expressed as means ± SD, **P < 0.01

The remarkable up‐regulation of HOTTIP in HSCs urged us to determine the underlying biological role of HOTTIP knockdown in liver fibrogenesis. Knockdown of HOTTIP markedly reduced HOTTIP expression relative to the Ad‐shCtrl (Figure 2A). Primary HSCs at day 4 were transduced with Ad‐shHOTTIP. As indicated in Figure 2B,C, the mRNA levels of Col1A1 and α‐SMA were decreased by 72% and 66% in Ad‐shHOTTIP cells, respectively, compared with Ad‐shCtrl cells. Consistently, the protein levels of type I collagen and α‐SMA were down‐regulated by 56% and 48% in Ad‐shHOTTIP cells, respectively (Figure 2D,E). Additionally, staining for type I collagen and α‐SMA presented the decreased levels of red fluorescence after Ad‐shHOTTIP treatment (Figure 2G,H). Then we examined whether HOTTIP down‐regulation inhibits proliferation of HSCs using an Edu incorporation assay. As indicated in Figure 2F, cells transduced with Ad‐shHOTTIP exhibited a remarkable down‐regulation in HSC proliferation in comparison with those transduced with Ad‐shCtrl. Collectively, our data indicated that down‐regulation of HOTTIP can repress the activation and proliferation of HSCs.

### 3.3 HOTTIP interacts with miR‐150

**Figure 3:**
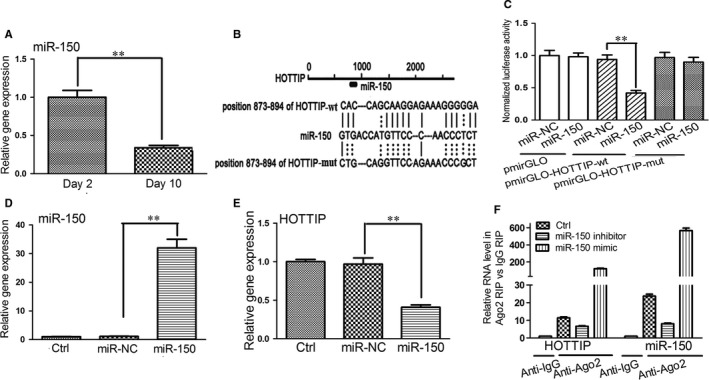
Interaction between HOTTIP and miR‐150. (A) Expression of miR‐150 was detected in primary HSCs at day 2 and day 10. (B) Schematic of the miR‐150 binding site in the HOTTIP. (C) Interaction between the HOTTIP and miR‐150 in HEK293T cells was analysed using reporter gene assay. (D) Expression of miR‐150 was detected in primary HSCs after miR‐150 treatment. (E) Expression of HOTTIP was detected in primary HSCs after miR‐150 treatment. (F) RIP assay was used to present the association of HOTTIP and miR‐150 with Ago2. Fold enrichment in Ago2 compared with IgG immunoprecipitates was used to indicate relative expression of HOTTIP and miR‐150. Results are expressed as means ± SD, **P < 0.01

Our previous study indicates that miR‐150 is decreased in activated LX‐2 cells.^14^ Here, we detected the expression of miR‐150 in mouse HSCs by qRT‐PCR. There was a 66% reduction in the expression of miR‐150 in primary mouse HSCs at day 10 compared with day 2 (Figure 3A), consistent with the previous report.^28^ Recently, some miRNAs have been reported to directly inhibit lncRNA expression.^29^, ^30^ Here, we explored whether miR‐150 can suppress HOTTIP expression. The online software RNA22 was employed to predict that HOTTIP contains a target of miR‐150 (Figure 3B). Next, we applied the luciferase assays to confirm whether miR‐150 binds to HOTTIP. As shown in Figure 3C, miR‐150 mimics effectively reduced the luciferase activity in pmirGLO‐HOTTIP‐wt compared with the miR‐NC. However, mutating the seed regions for miR‐150 almost abolished its luciferase activity. Then, we explored the effect of miR‐150 on the expression of HOTTIP in HSCs. In comparison with the miR‐NC, delivery of miR‐150 mimics remarkably increased the level of miR‐150 (Figure 3D). Consistently, miR‐150 mimics resulted in the 58% reduction in HOTTIP expression relative to the miR‐NC (Figure 3E). Taken together, our data indicates that HOTTIP is a target of miR‐150.

We next set out to investigate whether HOTTIP binds with miR‐150. It has been reported that miR‐21 and GAS5 can inhibit each other via the RNA‐mediated silencing mechanism. We inferred that HOTTIP and miR‐150 might suppress each other in a similar way. miRNAs are present in the form of miRNA ribonucleoprotein complexes (miRNPs) including Ago2, the major element of the RNA‐induced silencing complex. To validate whether HOTTIP interacts with miRNPs, RIP assay was conducted on primary HSCs using anti‐Ago2 antibody. HOTTIP and miR‐150 were enriched by 10.4‐fold and 22.7‐fold in the Ago2 pellet compared with IgG pellets, respectively. After miR‐150 overexpression or underexpression, expression of HOTTIP loaded onto the Ago2 pellet was enriched or diminished accordingly (Figure 3F). These findings suggest that HOTTIP is recruited to Ago2‐related miRNPs and likely through association with miR‐150.

### 3.4 HOTTIP controls SRF expression by competing for miR‐150

**Figure 4:**
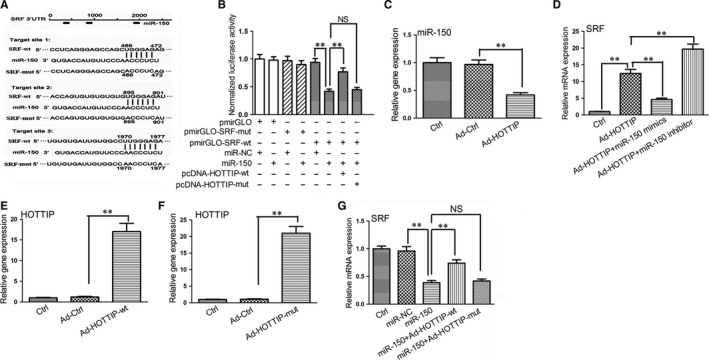
HOTTIP regulates SRF expression via sponging miR‐150. (A) Putative miR‐150 binding site in the 3′UTR of SRF mRNA are shown. (B) Luciferase activities of luciferase reporters containing the wild‐type or mutant SRF were detected in HEK293T cells after miR‐150 mimics together with or without pcDNA‐HOTTIP‐wt or pcDNA‐HOTTIP‐mut. (C) Expression of miR‐150 was detected in primary HSCs after Ad‐HOTTIP treatment. (D) The mRNA expression of SRF was detected in primary HSCs after Ad‐HOTTIP treatment, together with miR‐150 mimics or miR‐150 inhibitor. (E) Expression of HOTTIP was detected in primary HSCs after Ad‐HOTTIP‐wt treatment. (F) Expression of HOTTIP was detected in primary HSCs after Ad‐HOTTIP‐mut treatment. (G) The mRNA expression of SRF was detected in primary HSCs after treatment with miR‐150, together with or without Ad‐HOTTIP‐wt or Ad‐HOTTIP‐mut. Results are expressed as means ± SD, **P < 0.01

It has been reported that some lncRNAs can serve as a sponge by binding miRNAs, thereby abrogating miRNA‐induced inhibiting activity on the targets. Herein, we investigated whether HOTTIP can regulate target expression by competing for miR‐150. Among the putative miR‐150 targets, we selected SRF given that it is in control of transcription of α‐SMA and Col1A1. We found that SRF 3′UTR harbours three target sites of miR‐150 based on TargetScan software (Figure 4A). As shown in Figure 4B, compared with the miR‐NC, miR‐150 mimics remarkably reduced the luciferase activity (55% inhibition) of the pmirGLO‐SRF‐wt reporter. However, miR‐150 mimics did not decrease the luciferase activity in pmirGLO‐SRF‐mut, confirming that SRF is a direct target of miR‐150.

Our previous data have indicated that miR‐150 can inhibit the activation of HSCs by binding to Sp1 and Col4A4 in the human LX‐2 cells. However, bioinformatics analysis (TargetScan) showed that Sp1 is not the target of miR‐150 in mice. In addition, although Col4A4 is predicted to the target of miR‐150 in mice, the total context+ score is very low. To further validate the above predicted results, we analysed the mRNA expression of Sp1 and Col4A4 in primary mouse HSCs transfected with miR‐150 mimics. As expected, overexpression of miR‐150 did not decrease the mRNA expression of Sp1 and Col4A4 (Figure S1A,B).

Then we asked whether HOTTIP serves as a sponge to deter miR‐150‐induced degradation of SRF mRNA. Compared with the Ad‐Ctrl, HOTTIP overexpression decreased the level of miR‐150 (Figure 4C). Then we explored the effect of HOTTIP on the mRNA expression of SRF. As shown in Figure 4D, overexpression of HOTTIP increased the mRNA expression of SRF compared with the control. Compared with Ad‐HOTTIP alone, miR‐150 mimics reduced the mRNA expression of SRF, whereas miR‐150 inhibitor increased the mRNA expression of SRF. In addition, our results indicated that miR‐150‐induced reduction in the luciferase activity was rescued by HOTTIP‐wt overexpression. By contrast, exogenous expression of HOTTIP‐mut did not restore the decreased luciferase activity by miR‐150 (Figure 4B). Then we verified the above results in primary HSCs. As indicated in Figure 4E,F, overexpression of Ad‐HOTTIP‐wt or Ad‐HOTTIP‐mut notedly increased the expression of HOTTIP relative to the Ad‐Ctrl, respectively. Compared with the miR‐NC, miR‐150 decreased the mRNA expression of SRF (Figure 4G). However, overexpression of Ad‐HOTTIP‐wt but not Ad‐HOTTIP‐mut rescued the down‐regulation of SRF by miR‐150. Our results support the conclusion that lncRNA can sequester miRNA, thus stopping it from repressing its target.

### 3.5 HOTTIP promotes HSC activation through decreasing miR‐150 and increasing SRF

**Figure 5:**
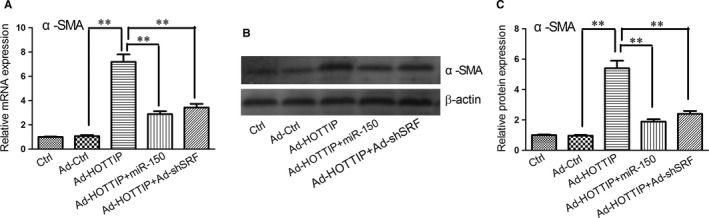
The increased expression of α‐SMA by HOTTIP overexpression was reversed by miR‐150 or SRF silencing. (A) Up‐regulation of α‐SMA mRNA by HOTTIP overexpression was alleviated by miR‐150 or SRF silencing. (B, C) Up‐regulation of α‐SMA protein by HOTTIP overexpression was alleviated by miR‐150 or SRF silencing. Results are expressed as means ± SD, **P < 0.01

Finally, we investigated whether miR‐150 and SRF are required for HOTTIP‐promoted HSC activation. As shown in Figure 5, HOTTIP overexpression induced a marked increase in the mRNA and protein expression of α‐SMA. However, up‐regulation of α‐SMA mRNA and protein expression by HOTTIP addition was attenuated by miR‐150 mimics or Ad‐shSRF, indicating that the antifibrotic effect of HOTTIP is dependent on miR‐150 and SRF.

## 4 DISCUSSION

In this study, we show the pathological implication of HOTTIP in facilitating the activation and proliferation of HSCs. Mechanistically, HOTTIP can function as a regulator of SRF expression through miR‐150 binding, which provides an intriguing approach for treating liver fibrosis.

Accumulating studies reveal that HOTTIP has been up‐regulated in a multitude of diseases.^31^, ^32^, ^33^ However, we failed to detect an aberrant expression of HOTTIP in fibrotic livers. It is likely that dysregulation of HOTTIP may occur particularly in a certain hepatic cell. As expected, the increased expression of HOTTIP was observed in activated HSCs, whereas no significant difference was found between hepatocytes isolated from oil‐treated mice and CCl_4_‐treated mice, suggesting that HOTTIP participates in the progression of liver fibrosis. Given that hepatocyte is the major cell type in the liver, we reason that invariability of HOTTIP in hepatocytes may conceal its alteration in HSCs.

HOTTIP has been reported to contribute to oncogenesis and tumour metastasis, thus identified as an oncogenic lncRNA. However, the role of HOTTIP in liver fibrosis has not been well‐characterized. To this end, we constructed adenoviral vectors expressing shRNA against mouse HOTTIP. Data from the present study demonstrated that HOTTIP silencing inhibits the activation and proliferation of HSCs, suggesting that HOTTIP plays a profibrogenic role in the progression of liver fibrosis.

Having shown the function of HOTTIP in promoting the progression of liver fibrosis, we next explore the underlying mechanism of HOTTIP. Dysregulations of lncRNAs and miRNAs have been shown to be in a variety of human diseases.^34^, ^35^ Some lncRNAs have been reported to act as competing endogenous RNAs (ceRNAs) via their competition for miRNA binding.^36^, ^37^ Here, we hypothesized that HOTTIP exerts its function in a similar way and sought for potential interactions with miRNAs. Based on bioinformatics analysis, HOTTIP was predicted the presence of a putative miR‐150 binding site. As expected, miR‐150 led to an obvious reduction in HOTTIP expression in HSCs. Luciferase reporter assay and RIP assay further indicated the interplay between HOTTIP and miR‐150. In addition, the decreased luciferase activity and mRNA expression of SRF by miR‐150 was partially reversed by Ad‐HOTTIP‐wt but not Ad‐HOTTIP‐mut, which supports the conclusion that ceRNA can sequester miRNA, thus protecting its target mRNA against suppression.

Our previous study demonstrates that miR‐150 can inhibit the activation of HSCs through targeting Sp1 and Col4A4. Here we identified SRF as the new target of miR‐150, which enlarges the repertoire of miRNA targets. Further study showed that miR‐150 and SRF are involved in the profibrogenic effect of HOTTIP, which implies that targeting the HOTTIP‐miR‐150‐SRF axis may signify a new therapeutic application in liver fibrosis.

There are some limitations in the current study. HOTTIP may sequester many miRNAs simultaneously and one miRNA has many targets. Consequently, “multiple‐to‐multiple” ceRNA interactions exists in the real cellular context instead of this simple “one‐to‐one” model. In addition, the biological role of HOTTIP in vivo has not yet to be investigated.

In summary, our experimental data suggest that HOTTIP can serve as the endogenous sponge to bind miR‐150 and then release its target SRF, thereby promoting the progression of liver fibrosis.

## CONFLICT OF INTERESTS

The authors confirm that there are no conflicts of interest.

## AUTHORS’ CONTRIBUTION

Fujun Yu and Zhiming huang designed the study; Jianjian Zheng and Yuqing Mao performed the research and wrote the manuscript; Peihong Dong analysed the data.
