# Supplementary figures and images for: Long noncoding RNA HOTTIP mediates SRF expression through sponging miR‐150 in hepatic stellate cells
Source: J Cell Mol Med. 2018 Dec 8;23(2):1572–80. doi: 10.1111/jcmm.14068 (PMC6349348; doi:10.1111/jcmm.14068)

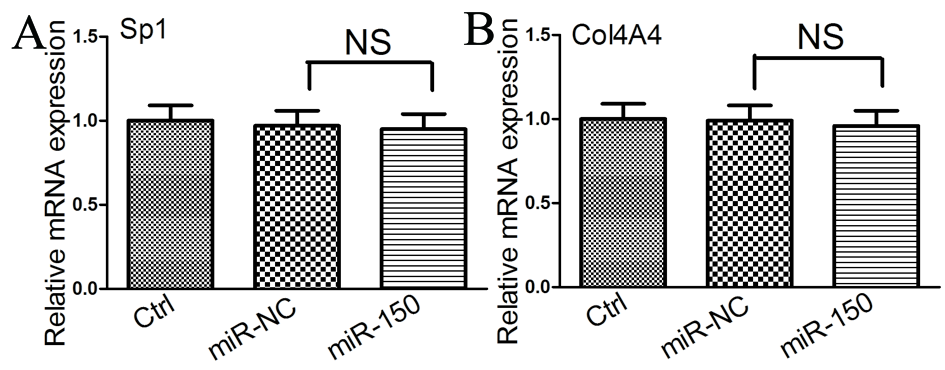

Supplement: Supplementary file 1 [file JCMM-23-1572-s001.tif]
